# Automated bolus advisor control and usability study (ABACUS): does use of an insulin bolus advisor improve glycaemic control in patients failing multiple daily insulin injection (MDI) therapy? [NCT01460446]

**DOI:** 10.1186/1471-2296-13-102

**Published:** 2012-10-13

**Authors:** David A Cavan, Ralph Ziegler, Iain Cranston, Katharine Barnard, Jacqueline Ryder, Claudia Vogel, Christopher G Parkin, Walter Koehler, Iris Vesper, Bettina Petersen, Robin S Wagner

**Affiliations:** 1 Royal Bournemouth Hospital, Castle Lane East, Bournemouth, BH7 7DW, UK; 2 Diabetes Clinic for Children and Adolescents, Mondstr. 148, 48155, Muenster, Germany; 3 Academic Department of Diabetes & Endocrinology Queen Alexandra Hospital, Portsmouth, PO6 3LY, UK; 4 University of Southampton, IDS Building, Southampton General Hospital, Tremona Road, Southampton, UK; 5 Internistisches Fachaerztezentrum, Rontgenstraffe 6-8, D-63225, Langen, Germany; 6 CGParkin Communications, 219 Red Rock Road, Boulder City, NV 89005, USA; 7 Walter Koehler, baseline statistics GmbH, Max-Joseph-Str, Mannheim, Germany; 8 Roche Diagnostics GmbH, Sandhofer Straße 116, 68305, Mannheim, Germany; 9 Roche Diagnostics, 9115 Hague Road, Indianapolis, IN, 46250, USA

**Keywords:** Insulin therapy, Multiple daily injections, Diabetes, Randomised, HbA1c, Psychosocial, Hypoglycaemia

## Abstract

**Background:**

People with T1DM and insulin-treated T2DM often do not follow and/or adjust their insulin regimens as needed. Key contributors to treatment non-adherence are fear of hypoglycaemia, difficulty and lack of self-efficacy associated with insulin dose determination. Because manual calculation of insulin boluses is both complex and time consuming, people may rely on empirical estimates, which can result in persistent hypoglycaemia and/or hyperglycaemia. Use of automated bolus advisors (BA) has been shown to help insulin pump users to more accurately meet prandial insulin dosage requirements, improve postprandial glycaemic excursions, and achieve optimal glycaemic control with an increased time within optimal range. Use of a BA containing an early algorithm based on sliding scales for insulin dosing has also been shown to improve HbA1c levels in people treated with multiple daily insulin injections (MDI). We designed a study to determine if use of an automated BA can improve clinical and psychosocial outcomes in people treated with MDI.

**Methods/design:**

The Automated Bolus Advisor Control and Usability Study (ABACUS) is a 6-month, prospective, randomised, multi-centre, multi-national trial to determine if automated BA use improves glycaemic control as measured by a change in HbA1c in people using MDI with elevated HbA1c levels (#62;7.5%). A total of 226 T1DM and T2DM participants will be recruited. Anticipated attrition of 20% will yield a sample size of 90 participants, which will provide #62;80% power to detect a mean difference of 0.5%, with SD of 0.9%, using a one-sided 5% *t*-test, with 5% significance level. Other measures of glycaemic control, self-care behaviours and psychosocial issues will also be assessed.

**Discussion:**

It is critical that healthcare providers utilise available technologies that both facilitate effective glucose management and address concerns about safety and lifestyle. Automated BAs may help people using MDI to manage their diabetes more effectively and minimise the risk of long-term diabetes related complications. Findings from a recent study suggest that BA use positively addresses both safety and lifestyle concerns; however, randomised trials are needed to confirm these perceptions and determine whether bolus advisor use improves clinical outcomes. Our study is designed to make these assessments.

**Trial registration:**

NCT01460446

## Background

Large clinical trials have shown that intensive management of glycaemia and other vascular risk factors can prevent or delay the development of microvascular and macrovascular complications in both type 1 diabetes (T1DM) and type 2 diabetes (T2DM) [1-4]. Achieving optimal glycaemic control in people with T1DM [1,2] and advanced T2DM [5] often requires intensive insulin therapy, which involves either use of insulin pumps or multiple daily insulin injections (MDI) [1,2,5]. Such therapies divide insulin doses into basal, prandial and corrective elements, which can be adjusted independently to achieve optimal results. Despite the benefits of intensive diabetes management, many people with T1DM and insulin-treated T2DM do not follow and/or adjust their insulin regimens as needed [6-9]. A recent survey of 331 people with T1DM showed that 64% of the participants assessed their prandial insulin need inappropriately [10].

A significant obstacle to intensive insulin management is fear of hypoglycaemia [11-14], which has detrimental effects on people’s willingness to effectively manage their diabetes, particularly in terms of appropriate insulin dosing (e.g., under-dosing of insulin to avoid hypoglycaemia). This can lead to poor metabolic control and subsequent poor health outcomes [14]. Other key contributors to treatment non-adherence are lack of self-efficacy and difficulties associated with insulin dose determination. Calculation of an insulin dose is a complex process that must take numerous factors into account, such as the current preprandial glucose level, grams of carbohydrate (CHO) to be ingested, insulin sensitivity, insulin-to-CHO ratio, and active insulin on board. Further, poor glycaemic control has been correlated with poor numeracy skills [15]. This may lead to an inability to count carbohydrates, errors in interpreting blood glucose (bG) results to determine correction doses and inaccuracies in calculating insulin doses based on insulin-to-CHO ratios.

Although many insulin pumps now feature automated bolus advisors, which automatically calculate bolus insulin dosages to cover carbohydrate (CHO) intake and address out-of-range bG levels based on individualised insulin parameter estimates, people using MDI therapy must perform these dosage calculations manually. However, because manual calculation of insulin boluses is both complex and time consuming, people may rely on empirical estimates, which can result in persistent hypoglycaemia and/or hyperglycaemia [16,17]. In addition, manual bolus calculation does not take into account the effect of the active insulin that remains from the initial bolus (insulin-on-board), which creates a high potential for errors, particularly when determining a correction bolus.

Studies have demonstrated that use of automated bolus calculators helps insulin pump users more accurately meet prandial insulin dosage requirements, improve postprandial glycaemic excursions, and achieve optimal glycaemic control with an increased time within target range [18,19]. It is, indeed, possible that such automated calculations may be a major source of the perceived benefits of pump therapy for many patients. For example, a study by Garg and colleagues [20] showed that use of a bolus advisor containing an early algorithm based on sliding scales for insulin dosing in people using MDI resulted in improved HbA1c levels. Results from a recent survey [21] suggest that use of an automated bolus advisor may reduce fear of hypoglycaemia, increase confidence in bolus calculation, improve ability to control bG levels and achieve glycaemic goals, create a sense of increased flexibility in lifestyle, and improve overall well being. In a small pilot study, Schmidt and colleagues [22] found that automated bolus advisor use, in conjunction with training in CHO counting and MDI therapy, improves treatment satisfaction. However, to date, no large randomised trials have been conducted to determine whether use of an automated bolus advisor can improve glycaemic control and promote greater adherence to therapy in people treated with MDI therapy.

We hypothesised that, in addition to reducing HbA1c, use of an automated bolus advisor in people treated with MDI therapy can increase the time bG stays within the target range, reduce the magnitude of post-prandial excursions, reduce the frequency/severity of hypoglycaemia, and improve psychosocial outcomes, including treatment satisfaction, social functioning and factors important to quality of life. We designed a study to test this hypothesis, using a new automated bolus advisor system that integrates bolus calculation into a bG meter.

## Methods/design

The Automated Bolus Advisor Control and Usability Study (ABACUS) is a 6-month, multi-center, multi-national, prospective, randomised, controlled study to assess the impact of automated bolus advisor use on clinical and psychosocial outcomes in poorly controlled (#62;7.5% HbA1c), MDI-treated T1DM and T2DM participants. The trial will be conducted at approximately 30 sites in the UK and Germany. All sites will have prior experience working with the bolus advisor prior to study initiation. All clinical investigators from the sites involved will be given detailed educational input regarding the device settings and adjustment principles (either using the automated bolus advisor or using more traditional ‘paper-copy’ (e.g., insulin-to-carbohydrate ratio/insulin sensitivity factor) at centralised study training sessions. The study protocol was approved by the National Research Ethics Service (Redditch, UK) and Ethik-Kommission der Ärztekammer Westfalen-Lippe und der Medizinischen Fakultät der Westfälischen Wilhelms-Universität Münster (Münster, Germany) and is in compliance with the Helsinki Declaration [23].

The primary outcome of the study is to determine if use of an automated bolus advisor is associated with a significant reduction in HbA1c over 6 months, compared with a group having the same diabetes educational background but with no access to an automated bolus advisor. The goal is to decrease HbA1c by #62;0.5%. Secondary outcomes include: 1) change in time spent within bG target range; 2) frequency and severity of hypoglycaemia; 3) change in magnitude of postprandial glucose excursions; 4) change in glycaemic variability parameters; 5) frequency of bolus advisor use; 6) frequency of participants’ adjustments to proposed bolus amounts; 7) self-monitoring of blood glucose (SMBG) test frequency; 8) change in participants’ therapy adherence and use of rule sets; and 9) participant self-care behaviours, and psychosocial outcomes including treatment satisfaction, social functioning and factors important to quality of life.

### Participants

The study will recruit 226 T1DM and T2DM patients who have been using MDI therapy for at least 6 months. Participants will be enrolled and randomised to the experimental (EXP) or control (CNL) groups; 113 participants in each group. This will lead to a total of 90 participants completing each arm at 6 months, assuming a drop-out rate of 20%.

Key inclusion criteria are: ≥18 years of age; T1DM or T2DM treated with MDI therapy for ≥6 months; HbA1c #62;7.5%; adjusts meal insulin doses based on CHO content of meal; and has completed CHO training within last 2 years. Key exclusion criteria are: NPH or pre-mixed insulin; treated with anti-diabetic agents (except metformin); use of fixed-dose therapy; or use of sliding scale insulin doses determined exclusively on specific bG results. The complete list of inclusion and exclusion criteria is presented in Table 1.

**Table 1:** Inclusion and exclusion criteria

**Inclusion Criteria:**	
· Must be 18 years of age or older	
· Diagnosed with Type 1 or Type 2 diabetes	
· Recent HbA1c #62;7.5% (measured within the last 6 weeks at local laboratory)	
· Using MDI therapy for at least 6 months consisting of 1–2 injections per day of long-acting basal insulin (Lantus® or Detemir®) and at least 2 injections per day of regular or rapid-acting analog insulin for meal coverage	
· Participant adjusts meal insulin doses based on carbohydrate content of meals	
· Participant with Type 2 diabetes may be on stable metformin therapy (therapy unchanged during 3 months prior to study)	
· Participant has been in Investigator's practice for at least 3 months; however may have been seen by another physician in the practice	
· Participant has completed carbohydrate (CHO) training within the last 2 years	
**Exclusion Criteria:**	
· On a therapy regimen that conflicts with study:	
· NPH or pre-mixed insulin	
· Oral anti-diabetic agents, with the exception of metformin	
· Injectable anti-diabetic agents other than long-acting insulin and rapid-acting insulin analogs or regular insulin (e.g., fixed dose therapy)	
· Use of sliding scale insulin therapy that determines insulin dosages based exclusively on specific bG results	
· Participated in another interventional trial within 6 weeks prior to study	
· Diagnosed with any clinically significant infectious disease or major organ system disease, such as gastroparesis or renal disease (at Investigator's discretion)	
· Used systemic oral or inhaled steroids for more than 7 days within the last 3 months	
· On Chemotherapy or Radiation therapy (self-reported)	
· Pregnant or lactating or is currently planning a pregnancy	

Participants will be identified and recruited from the investigators’ established patient population or from within the patient population of other physicians within their group practice using the inclusion/exclusion criteria. Participants who agree to take part in the study and meet all of the inclusion but none of the exclusion criteria will be asked to sign the approved Informed Consent Form for the study.

### Design

**Figure 1:**
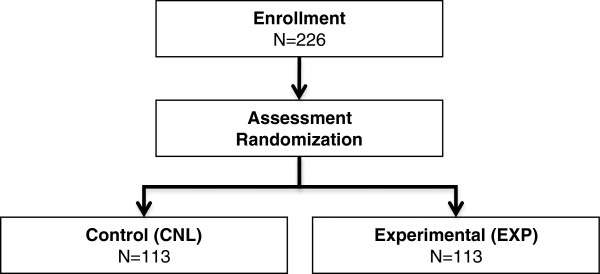
CONSORT Diagram.

Participants will be randomised (1:1) by participant code number to a control group (CNL) or to bolus advisor use (EXP) where they will utilise an automated bolus advisor to determine prandial and correction insulin doses. Both study groups will receive intensive diabetes management with frequent assessment and adjustment of insulin therapy parameters. The study design is shown in Figure 1. Participants from both arms will be evaluated with the same scales and measures at the same time intervals over 6 months.

### Procedure

EXP participants will utilise the Accu-Chek® Aviva (Roche Diagnostics, Indianapolis, USA), which incorporates an automated bolus advisor into a blood glucose meter. Users can obtain prandial and correction bolus recommendations based upon a current blood glucose value, planned carbohydrate intake and individualised therapy parameters stored in the device. The device automatically calculates the appropriate bolus for the user and stores blood glucose and meal information in an electronic diary. A previous study [24] showed that the automated bolus advisor device can assist people in achieving desired postprandial glucose control without significant hypoglycaemia.

Physicians and staff in both arms will be informed about the clinical investigational plan, including the rationale for the study, design of the study protocol, subject-related procedures, and use of evaluation questionnaires. EXP physicians and staff will receive training on using the automated bolus advisor device to set insulin parameters and download SMBG and insulin dosing data.

Approximately 50-60% of the sites (those with prior experience of CGM use) will utilise a blinded continuous glucose monitoring (CGM) device (DexCom Seven® Plus, DexCom, Inc., San Diego, California, USA), with data uploaded directly to a designated secure server. Clinicians and participants will not have access to this CGM data for the duration of the study, which will be analysed centrally to contribute to the secondary end-points of the study.

### Visit schedule

**Figure 2:**
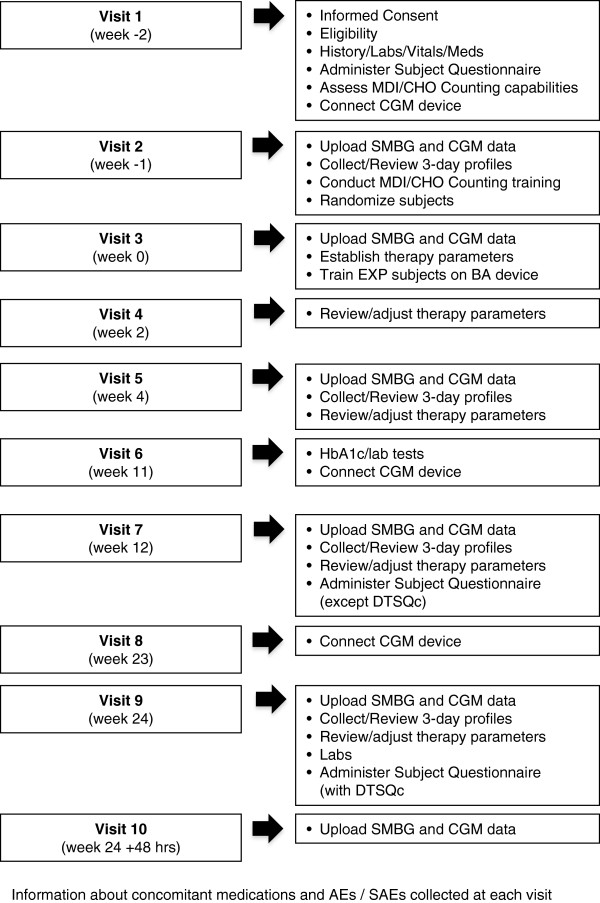
Study Visit Schedule.

The 6-month study will consist of a screening and training period of approximately 2 weeks ± 5 days and an intervention period of approximately 24 weeks. An optional follow-up patient survey by the investigator will be administered after an additional 26 weeks following Visit 10 (End of Study) (Figure 2).

#### Visit 1: screening and training (week – minus 2)

At Visit 1, investigators will confirm participants’ eligibility for the study (based on inclusion/exclusion criteria), obtain written informed consent, record demographic information, collect relevant medical history/lifestyle information, document all current medications, perform physical examinations, measure and record weight, height, blood pressure, heart rate, collect blood and urine samples for laboratory tests (HbA1c, urinalysis, chemistry panel with lipids, full blood count [FBC]) and perform pregnancy testing (for women of child bearing age). Participants will be asked to complete a questionnaire that incorporates the questions from standard psychometric instruments (See *Measures* section).

Investigators will use standardised worksheets to assess participants’ knowledge and skills relevant to MDI therapy and CHO counting. Selected Dose Adjustment For Normal Eating (DAFNE) plates will also be used in the CHO counting assessment. Deficits in MDI therapy and CHO counting skills will be documented and addressed during Visit 2.

Participants in both groups will initially receive a blood glucose meter (Accu-Chek® Nano blood glucose meter, Roche Diagnostics, Indianapolis, Indiana, USA) and will be thoroughly trained in its operation. Where applicable, investigators will connect the CGM device to subjects and provide training.

Participants will be instructed to generate 7-point glycaemic profiles (preprandial/2-hours postprandial at all meals, and bedtime) over 3 consecutive days and document their results on a standardised form that will be provided. Using the form, participants will also document details of their meals (including number of CHOs), physical exercise (intensity and duration), basal insulin doses, prandial bolus doses, and correction bolus doses over the 3-day testing period. CGM participants will be instructed to wear their device until Visit 2. This will be done in addition to completing their 3-day, 7-point glycaemic profiles.

#### Visit 2: randomisation and intensive monitoring (week – minus 1)

At Visit 2, investigators will collect participants’ bG meters, completed 3-day glycaemic profiles and CGM devices (if applicable). Investigators will upload bG meter data via the research version of the Accu-Chek® SmartPix device (Roche Diagnostics, Indianapolis, Indiana, USA). When applicable, CGM data will also be uploaded to the server. Blood glucose data will be downloaded to clinic software, using either the commercial Accu-Chek® Smart Pix device or Accu-Chek® 360˚ View diabetes management system software (Roche Diagnostics, Indianapolis, Indiana, USA), and investigators will prepare printouts to review with each participant. New adverse events (AEs) and significant adverse events (SAEs), or changes in ongoing AEs/SAEs since the last visit, will be assessed and recorded; participants’ concomitant medications will be updated.

Investigators will then conduct MDI and CHO counting training, which will be individualised to address each participant’s knowledge and/or skills deficits as identified during the MDI therapy and CHO counting assessments performed at Visit 1. Standardised checklists will be used to document participant competencies. Participants must be competent in both MDI therapy and CHO counting knowledge in order to continue in the study. Following this training, participants will be randomised to the control group (CNL) for usual care or to bolus advisor use (EXP). EXP participants will be given automated bolus advisor training materials for review at home.

#### Visit 3: training and therapy initiation (week – 0)

At Visit 3, investigators will collect participants’ bG meters, upload bG meter data to the secure server; download meter data to clinic software, and prepare printouts of bG data to review with participants. New AE/SAEs or changes to ongoing AE/SAEs since the last visit will be assessed and concomitant medications updated. Investigators will use the therapy parameter cards and instruct participants on how to use their parameters.

EXP participants will be given their automated bolus advisor device (Accu-Chek® Aviva Expert bG meter, Roche Diagnostics, Indianapolis, Indiana, USA) and instructed to discontinue use of their current bG meter. Investigators will conduct 1-hour training sessions in automated bolus advisor device use and update the devices with each participant’s new therapy parameters.

At the conclusion of the visit, all participants will receive SMBG supplies and a logbook with a 3-day glycaemic profile form. Participants will be instructed to complete the 3-day profiles one week prior to Visit 5.

#### Visit 4: telephone follow-up (week – 2)

Investigators will have the option to conduct Visit 4 as either a clinic visit or via telephone. New AE/SAEs or changes to ongoing AE/SAEs since the last visit will be assessed, concomitant medications updated, and therapy parameters will be adjusted as needed. Investigators will schedule the next visit (Visit 5) and remind participants to complete their 3-day glycaemic profile prior to that visit.

#### Visits 5 through 9 (weeks – 4 through 24)

At Visits 5 through 9, investigators will collect participants’ bG meters, upload bG meter data to a secure server; download meters to clinic software, and prepare printouts of bG data to review with participants. New AE/SAEs or changes to ongoing AE/SAEs since the last visit will be assessed and concomitant medications will be updated. At Visits 5, 7 and 9, participants’ 3-day glycaemic profiles and historic data will be collected and reviewed to adjust therapy parameters (if needed). If parameters are adjusted, participants’ automated bolus advisor devices will be updated with the new parameters. HbA1c samples will be collected at Visits 6 and 9. Additional laboratory samples will be obtained at Visit 9 for chemistry profile and lipid profile, FBC, perform dipstick urinalysis. At Visits 7 and 9, 5-day CGM data will be uploaded to the secure server. Patient questionnaires will be administered at Visits 7 and 9. Final CHO counting and MDI therapy assessments will be conducted at Visit 9.

#### Visit 10: telephone follow-up (week – 24 +48 hours)

At Visit 10, investigators will call each participant 48 hours after Visit 9 to assess and record new AE/SAEs or changes to ongoing AE/SAEs since their last visit.

#### Optional follow-up (26 weeks after Visit 10)

Investigators have the option to schedule an additional clinic visit approximately 26 weeks after Visit 10 to obtain participants’ most recent HbA1c value (performed locally as part of routine practice) and collect information about participants’ use of the automated bolus advisor device. This would include downloading data from the automated bolus advisor and documenting participants’ current insulin parameters.

### Measures

#### Primary endpoint

HbA1c: The primary endpoint of the study is glycaemic control, as assessed by change in HbA1c from baseline over 6 months specifically, looking at number of patients who achieved #62;0.5% HbA1c reduction at study end. Blood samples will be collected at screening and Visits 6 (week 11) and 9 (week 24). HbA1c analysis will be conducted by a central laboratory, using HPLC methodology (VII Turbo haemoglobin testing system, Bio-Rad Laboratories, Hercules, California, USA).

#### Secondary endpoints

*Time within Target Range:* CGM data (from 50-60% of participants) will be used to assess the change in time spent within the target bG range of 72–180 mg/dl from baseline to study end. CGM data will be collected at Visits 2 (week minus 1), 7 (week 12), and 9 (week 24).

*Other Glycaemic Measures:* Glucose data from 3-day glycaemic profiles, derived from uploaded SMBG and CGM data (when applicable) will be used to assess: 1) frequency of hypoglycaemia (within 50–72 mg/dl) and severe hypoglycaemia (<50 mg/dl with associated symptoms), overall, and by defined time blocks (12:00 a.m. to 5:00 a.m., 5:00 a.m. to 10:30 a.m., 10:30 a.m. to 4:30 p.m., 4:30 p.m. to 12:00 a.m.); 2) total number of SMBG tests and average number of SMBG tests per day and by the defined time blocks; 3) percentage of all values, preprandial values (60–0 minutes prior to meal) and postprandial values (60–180minutes after start of meal) within defined bG ranges (<50 mg/dl, 50–72 mg/dl, 72–180 mg/dl, 180–300 mg/dl, #62;300 mg/dl); 4) mean bG across the 3-day profiles and for each of the 7 time points; 5) standard deviation (SD) and coefficient of variation across the 3-day profiles; 6) Mean and SD differences between preprandial and postprandial bG values over each meal in the 3-day profiles; 7) mean amplitude of glycaemic excursions (MAGE), low bG index (LBGI), high bG index (HBGI), bG risk index (BGRI) and risk for hypoglycaemia and hyperglycaemia across the 3-day profiles. The 3-day glycaemic profile data will be collected at Visits 2 (week minus 2), 5 (week 4), 7 (week 12) and 9 (week 24).

*Automated Bolus Advisor Use and Participants’ Adjustments (EXP Group):* The frequency of participants’ bolus advisor use and subsequent adjustments based on proposed bolus amounts will be derived from data uploads of the automated bolus advisor device. Analyses will include: average number of times per day participants sought advice for mealtime and correction bolus calculation; average number of times per day participants accepted the automated bolus advisor advice; average number of insulin doses adjusted up or down per day; average number of times participants accepted the automated bolus advisor responses and adjusted boluses within specific time periods (a.m., p.m., after 10 p.m.); and differences between the average number of adjusted mealtime boluses and adjusted correction boluses.

*Participant Adherence/Use of Rule Sets:* Changes in participants’ therapy adherence to and use of rule sets in EXP participants will be derived from downloaded data obtained from automated bolus advisor devices. Adherence in CNL participants will be derived from documented therapy parameters and participants’ diaries, which are included in 3-day glycaemic profile forms. These assessments will be used to determine if participants’ adherence/use of rule sets correlates with improvement in glycaemic control. Analyses will include: average number of correctly and incorrectly used insulin-to-carbohydrate (I:CHO) rules per day for CHO intake; average number of correctly and incorrectly used insulin sensitivity factor (ISF) rules per day for bG adjustment; and number of changed I:CHO and ISF parameters compared to therapy parameters at study start (Visit 3). Data will be collected at Visits 2 (week minus 2), 5 (week 4), 7 (week 12) and 9 (week 24).

*CHO Counting:* Changes from baseline in participants’ ability to accurately count CHO will be assessed using the DAFNE plate assessment scores from Visit 1 (week minus 2) and 9 (week 24). This assessment will be used to determine if participants’ CHO counting skills correlates with improvement in glycaemic control.

*Psychosocial Outcomes*: Psychosocial outcomes including treatment satisfaction, social functioning and factors important to quality of life will be assessed using the participant questionnaire, which incorporates questions from validated psychometric instruments, as well as commonly used survey questions, to assess depression, diabetes-specific distress, diabetes self-efficacy, treatment satisfaction, health outcomes and fear of hypoglycaemia. Participant questionnaire items include: Patient Health Questionnaire depression scale (PHQ-8) [25]; Problem Area in Diabetes (PAID) [26]; Hypoglycaemia Fear Scale (HFS –II) [11];Diabetes Treatment Satisfaction Questionnaire (DTSQs, [baseline] and DTSQc [change]) [27];Gold Scale [28];and EuroQol-5 Dimensions-5 Levels measure (EQ-5D-5L) [29] (Table 2). The questionnaires will be administered at Visits 1 (week minus 2), 7 (week 12) and 9 (week 24); however, the DTSQs and DTSQc portions will be administered only at Visits 1 and 9.

**Table 2:** Participant Questionnaire components

**Instrument**	**Description**	**Scoring**
*Patient Health Questionnaire 8 (PHQ-8)*[25]	Self-report measure of depression	Each of 8 items scores from 0=‘not at all’ to 3=‘nearly every day’ Major depressive disorder (PHQ-8): ≤ 4 – no significant symptoms, 5 to 9 – mild symptoms, 10 to 14 – moderate symptoms, 15 to 19 – moderately severe symptoms, ≥ 20 – severe symptoms
*Problem Area in Diabetes (PAID)*[26]	Covers a range of emotional states frequently reported in diabetes. It is primarily a measure of diabetes-specific emotional distress	Each of 20 items scores from 0=‘not a problem’ to 4=‘serious problem’ PAID summary score: 0 to 39 – no distress, 40 to 59 – mild distress 60 to 79 – moderate distress 80 to 100 – severe distress
*HFS II (Hypoglycaemia Fear Scale)*[11]	Provides an assessment of an individuals’ fear of hypoglycaemia both overall and in terms of behaviour and worry. IUsed to assess both fear of hypos and maladaptive behaviours to avoid them.	Each of 33 items scores from 0=‘never’ to 4=‘always’ Score for frequency/severity of avoidance behaviour: 0 to 36 – no/slight avoidance behaviour: 37 to 48 – moderate avoidance behaviour: 49 to 60 – frequent/severe avoidance behaviour Score for severity of worry: 0 to 43 – no/slight worry: 44 to 57 – moderate worry: 57 to 72 – frequent/severe worry
*Diabetes Treatment Satisfaction Questionnaire (baseline) DTSQs and DTSQc (change)*[27]	Baseline version measures patient satisfaction with diabetes treatment Change version developed to overcome ceiling effects with treatment satisfaction	DTSQs -- Each of 6 items scores from 0=‘very bad’ to 6=‘very good’ DTSQc -- Each of 6 items scores from −3=‘much worse now’ to 3=‘much better now’)
*Gold Scale*[28]	Categorises awareness of having reduced awareness of hypoglycaemia in patients with diabetes.	One item with score from 1=‘always aware’ to 7=‘never aware’ Impaired awareness if the Gold scale score is ≥4
EuroQol-5 Dimensions-5 Levels measure *(EQ-5D-5L)*[29]	Measures health outcomes and is applicable to a wide range of health conditions and treatments	Each of the 5 items score from 1=‘no problems’ to 5=‘unable’) EQ-5D health assessment scores from 0=‘worst health’ to 100=‘best health’)

All scales and measures used in the study will contain participant codes rather than participant identifiers. Participant identifiers will be restricted to their clinicians and senior investigators.

### Statistical analysis

#### Populations for analysis

The Intent to Treat (ITT) population is defined as all eligible participants who participated in Visit 1 (screening) and Visit 2 (randomisation); all efficacy and safety analyses will be performed on this population. The per protocol population (PP) will include all participants of the ITT group who completed all scheduled visits without any major protocol deviations. Participants must complete at least 80% of all bG measurements of the 3-day glycaemic profile form to be included in the PP analysis.

#### Primary outcome analysis

Anticipated attrition of 20% will yield a sample size of 180 (90 per study group), which will provide #62;80% power to detect a mean difference of 0.5% in change in HbA1c, with SD of 0.9%. The absolute and relative change in HbA1c from baseline to study end for the CNL and EXP groups will compared using the two-sample *t*-test for the pooled ITT population. These tests will be performed at the one-sided 5% level of significance for rejecting the null hypothesis of no difference or a difference in favor of the CNL group. An analysis of covariance with the binary covariables (study group, sex and diabetes type) and the continuous baseline covariables (HbA1c, age and CHO counting assessment score) will be performed for the absolute and relative HbA1c change. Additional separate analyses of (co)variance will be performed using two explanatory variables (study group and one adjusting covariable) for the above mentioned covariables and other baseline covariables (e.g., country, center, time since diabetes diagnosis, existing diabetes related diseases, time since MDI start, bG measures from SMBG at baseline, frequencies of hypoglycaemia at baseline, laboratory parameters and, summary scores from psychosocial questionnaires) for the pooled groups. The one-sided 95% confidence intervals of the HbA1c change for CNL and EXP groups and the difference between the groups will also be calculated for the pooled groups.

Summary statistics (N, mean, median, SD, lower and upper quartile, minimum and maximum) will be provided for the observed HbA1c values by study group for each applicable scheduled visit (Visits 1, 6 and 9). Graphical techniques will be used to display the HbA1c change over time.

Pearson correlation coefficients will be computed for the HbA1c values at Visits 1, 6, and 9 and the absolute and relative HbA1c changes from baseline to study end vs. demographic and clinical variables; bG measures from SMBG and CGM scores for each collection visit, other laboratory parameters, frequencies of hypoglycaemia, CHO scores; and summary scores from psychosocial questionnaires. For all of these covariables, the correlation coefficients for their changes from baseline vs. the HbA1c parameters will be computed, too. Additionally, the correlation coefficients for the HbA1c parameters vs. bolus advisor parameters will be computed for the EXP group.

#### Secondary outcome analyses

Statistical analysis of change in time spent within target range will be performed according to the same schemata used for the primary outcome. Other secondary outcome variables will be compared descriptively by study groups for each scheduled visit and for changes between visits. Continuous variables, including scores from the questionnaires will be summarised using N, mean, SD, median, lower and upper quartile, minimum and maximum; categorical variables will be summarised using counts and percentages of participants in each category. Two-sample t-tests in the case of continuous variables and chi-square tests in the case of categorical variables will be performed for group comparisons. Graphical techniques will be used to display changes over time, if appropriate. Pearson correlation coefficients will be computed between all variables of interest.

## Discussion

Optimal diabetes management is not achieved by many people with T1DM or insulin-treated T2DM who do not adjust their insulin regimens as needed [6-9]. This can lead to poor metabolic control and subsequent poor health outcomes [14]. Inadequate management of glycaemia within this population is complex and often the result of many factors, including: fear of hypoglycaemia [11-14]; lack of self-efficacy and difficulty with insulin dose adjustment; and poor numeracy skills [15].

Many of today’s insulin pump systems now feature automated bolus advisor technology, which calculates individualised bolus insulin dosages to cover carbohydrate (CHO) intake and address out-of-range bG levels. Studies have shown that use of automated bolus advisor technology among insulin pump users facilitates accurate determination of prandial insulin dosages, reduces postprandial excursion, and reduces hypoglycaemia, leading to improved glycaemic control [18,19].

Our study is designed to assess the impact of automated bolus advisor use on glycaemic control, user adherence, self-care behaviours, treatment satisfaction, social functioning and factors important to quality of life. A potential limiation of our study design is the intensity of diabetes management provided to both groups, which may lead to significant improvements in all participants and, thus, limit between-group differences. A third, “pure” control arm (no intervention) would have been ideal to compare the intervention with real-world clinical care; however, this option was not feasible.

Given the benefits of tight metabolic control, it is critical that healthcare providers utilise available technologies that not only facilitate effective glucose management but also address concerns about safety and lifestyle, which can discourage adherence to therapy. Although a growing body of evidence suggests that automated bolus advisor use positively addresses safety and lifestyle concerns [21,22], large, randomised trials are needed to confirm these findings and assess the utility of automated bolus advisor use in improving intra-day and long-term glycaemic control. Our study is designed to make these assessments. Findings will be available in late 2012.

## Abbreviations

ABACUS: Automated Bolus Advisor Control and Usability Study; AE: Adverse event; BA: Bolus advisor; BG: Blood glucose; BGRI: Blood glucose risk index; CGM: Continuous glucose monitoring; CHO: Carbohydrate; CNL: Control group; DAFNE: Dose Adjustment for Normal Eating; DTSQc: Diabetes Treatment Satisfaction Questionnaire (change); DTSQs: Diabetes Treatment Satisfaction Questionnaire (baseline); EQ-5D-5L: EuroQol-5 Dimensions-5 Levels measure; EXP: Experimental group; FBC: Full blood count; HbA1c: Glycated haemoglobin; HBGI: High blood glucose index; HFS II: Hypoglycaemia Fear Scale); I:CHO: Insulin-to-carbohydrate ratio; ITT: Intent to treat; LBGI: Low blood glucose index; MAGE: Mean amplitude of glycaemia excursions; MDI: Multiple daily insulin injections; PAID: Problem Area in Diabetes; PHQ-8: Patient Health Questionnaire 8; PP: Per protocol; SD: Standard deviation; SAE: Significant adverse event; SMBG: Self-monitoring of blood glucose; T1DM: Type 1 diabetes; T2DM: Type 2 diabetes.

## Competing interests

Funding for the study and preparation of the manuscript was provided by Roche Diagnostics, Indianapolis, Indiana, USA. DC, RZ, IC, KB, JR, CV, CP, and WK serve as paid consultants to Roche Diagnostics for their involvement in the study design, study implementation and preparation of the manuscript. CP has worked as a consultant for Roche Diagnostics, Abbott Diabetes Care (Alameda, California, USA), Amylin Pharmaceuticals (San Diego, California, USA), Sanofi-Aventis (Bridgewater, New Jersey, USA), and DexCom. (San Diego, California, USA) IV, BP, RW are employees of Roche Diagnostics.

## Authors’ contributions

DC, RZ, IC, and KB had the original idea for the study and wrote the study proposal and protocol. DC, RZ, IC, KB, JR, CV, BP, IV and RW developed study measures and intervention. JR, CV, and CP developed subject instructional materials and provided training. IV is the study site coordinator. WK provided statistical analysis support. DC, RZ, IC, KB, CP and RW developed the manuscript, which was reviewed by IV and BP. All authors read and approved the final manuscript.

## Pre-publication history

The pre-publication history for this paper can be accessed here:

http://www.biomedcentral.com/1471-2296/13/102/prepub

## References

[B1] The Diabetes Control and Complications Trial Study Group The effect of intensive treatment of diabetes on the development and progression of long-term complications in insulin-dependent diabetes mellitus N Engl J Med 1993 329 14 977 986 8366922 10.1056/NEJM199309303291401

[B2] Nathan DM Cleary PA Backlund JY Genuth SM Lachin JM Orchard TJ Raskin P Zinman B Intensive diabetes treatment and cardiovascular disease in patients with type 1 diabetes N Engl J Med 2005 353 25 2643 2653 16371630 10.1056/NEJMoa052187 PMC2637991

[B3] Group UKPDS Intensive blood-glucose control with sulphonylureas or insulin compared with conventional treatment and risk of complications in patients with type 2 diabetes (UKPDS 33) Lancet 1998 352 9131 837 853 9742976

[B4] Holman RR Paul SK Bethel MA Matthews DR Neil HA 10-Year Follow-up of Intensive Glucose Control in Type 2 Diabetes N Engl J Med 2008 359 15 1577 1589 10.1056/NEJMoa0806470 18784090

[B5] Heinemann L Overcoming obstacles: new management options Eur J Endocrinol 2004 151 Suppl 2 T23 T27 15487981 10.1530/eje.0.151t023

[B6] Di Battista AM Hart TA Greco L Gloizer J Type 1 diabetes among adolescents: reduced diabetes self-care caused by social fear and fear of hypoglycemia Diabetes Educ 2009 35 3 465 475 10.1177/0145721709333492 19321802

[B7] Morris AD Boyle DI McMahon AD Greene SA MacDonald TM Newton RW Adherence to insulin treatment, glycaemic control, and ketoacidosis in insulin-dependent diabetes mellitus. The DARTS/MEMO Collaboration. Diabetes Audit and Research in Tayside Scotland. Medicines Monitoring Unit Lancet 1997 350 9090 1505 1510 10.1016/S0140-6736(97)06234-X 9388398

[B8] Smith CB Choudhary P Pernet A Hopkins D Amiel SA Hypoglycemia unawareness is associated with reduced adherence to therapeutic decisions in patients with type 1 diabetes: evidence from a clinical audit Diabetes Care 2009 32 7 1196 1198 10.2337/dc08-2259 19389817 PMC2699737

[B9] Cramer JA A systematic review of adherence with medications for diabetes Diabetes Care 2004 27 5 1218 1224 10.2337/diacare.27.5.1218 15111553

[B10] Ahola AJ Makimattila S Saraheimo M Mikkila V Forsblom C Freese R Groop PH Many patients with Type 1 diabetes estimate their prandial insulin need inappropriately J Diabetes 2010 2 3 194 202 10.1111/j.1753-0407.2010.00086.x 20923484

[B11] Cox DJ Irvine A Gonder-Frederick L Nowacek G Butterfield J Fear of hypoglycemia: quantification, validation, and utilization Diabetes Care 1987 10 5 617 621 10.2337/diacare.10.5.617 3677982

[B12] Irvine AA Cox D Gonder-Frederick L Fear of hypoglycemia: relationship to physical and psychological symptoms in patients with insulin-dependent diabetes mellitus Health Psychol 1992 11 2 135 138 1582382 10.1037//0278-6133.11.2.135

[B13] Polonsky WH Davis CL Jacobson AM Anderson BJ Hyperglycaemia, hypoglycaemia, and blood glucose control in diabetes: symptom perceptions and treatment strategies Diabet Med 1992 9 2 120 125 10.1111/j.1464-5491.1992.tb01747.x 1563245

[B14] Wild D von Maltzahn R Brohan E Christensen T Clauson P Gonder-Frederick L A critical review of the literature on fear of hypoglycemia in diabetes: Implications for diabetes management and patient education Patien Educ Couns 2007 68 1 10 15 10.1016/j.pec.2007.05.003 17582726

[B15] Cavanaugh K Huizinga MM Wallston KA Gebretsadik T Shintani A Davis D Gregory RP Fuchs L Malone R Cherrington A Association of numeracy and diabetes control Ann Intern Med 2008 148 10 737 746 18490687 10.7326/0003-4819-148-10-200805200-00006

[B16] Pickup J Keen H Continuous subcutaneous insulin infusion at 25 years: evidence base for the expanding use of insulin pump therapy in type 1 diabetes Diabetes Care 2002 25 3 593 598 10.2337/diacare.25.3.593 11874953

[B17] Bode BW Sabbah HT Gross TM Fredrickson LP Davidson PC Diabetes management in the new millennium using insulin pump therapy Diabetes Metab Res Rev 2002 18 Suppl 1 S14 S20 11921425 10.1002/dmrr.205

[B18] Klupa T Benbenek-Klupa T Malecki M Szalecki M Sieradzki J Clinical usefulness of a bolus calculator in maintaining normoglycaemia in active professional patients with type 1 diabetes treated with continuous subcutaneous insulin infusion J Int Med Res 2008 36 5 1112 1116 18831908 10.1177/147323000803600531

[B19] Gross TM Kayne D King A Rother C Juth S A bolus calculator is an effective means of controlling postprandial glycemia in patients on insulin pump therapy Diabetes Technol Ther 2003 5 3 365 369 10.1089/152091503765691848 12828818

[B20] Garg SK Bookout TR McFann KK Kelly WC Beatson C Ellis SL Gutin RS Gottlieb PA Improved glycemic control in intensively treated adult subjects with type 1 diabetes using insulin guidance software Diabetes Technol Ther 2008 10 5 369 375 10.1089/dia.2007.0303 18715213 PMC2979335

[B21] Barnard KD Parkin CG Young A Ashraf M Use of an automated bolus calculator reduces fear of hypoglycemia and improves confidene in dosage accuracy in patients with type 1 diabetes mellitus treated with multiple daily insulin injections J Diab Sci Tech 2012 6 1 144 149 10.1177/193229681200600117 PMC3320831 22401332

[B22] Schmidt S Meldgaard M Serifovski N Storm C Christensen TM Gade-Rasmussen B Norgaard K Use of an Automated Bolus Calculator in MDI-Treated Type 1 Diabetes: The BolusCal Study, a randomized controlled pilot study Diabetes Care 2012 35 984 990 10.2337/dc11-2044 22344610 PMC3329826

[B23] World Medical Association declaration of Helsinki Recommendations guiding physicians in biomedical research involving human subjects JAMA 1997 277 11 925 926 9062334

[B24] Zisser H Wagner R Pleus S Haug C Jendrike N Parkin C Schweitzer M Freckmann G Clinical performance of three bolus calculators in subjects with type 1 diabetes mellitus: a head-to-head-to-head comparison Diabetes Technol Ther 2010 12 12 955 961 10.1089/dia.2010.0064 21128842

[B25] Kroenke K Spitzer RL Williams JB The PHQ-9: validity of a brief depression severity measure J Gen Intern Med 2001 16 9 606 613 10.1046/j.1525-1497.2001.016009606.x 11556941 PMC1495268

[B26] Polonsky WH Anderson BJ Lohrer PA Welch G Jacobson AM Aponte JE Schwartz CE Assessment of diabetes-related distress Diabetes Care 1995 18 6 754 760 10.2337/diacare.18.6.754 7555499

[B27] Bradley C Bradley C The Diabetes Treatment Satisfaction Questionnaire (DTSQ) Handbook of Psychology and Diabetes: A Guide to Psychological Measurement in Diabetes Research and Practice 1994 Hardwood Academic, Chur, Switzerland 111 132

[B28] Gold AE Macleod KM Frier BM Frequency of severe hypoglycemia in patients with type I diabetes with impaired awareness of hypoglycemia Diabetes Care 1994 17 7 697 703 10.2337/diacare.17.7.697 7924780

[B29] Clarke P Gray A Holman R Estimating utility values for health states of type 2 diabetic patients using the EQ-5D (UKPDS 62) Med Decis Making 2002 22 4 340 349 12150599 10.1177/0272989X0202200412

